# Robotic approach for OHVIRA syndrome: case report of an early diagnosis in a prepubertal girl

**DOI:** 10.1097/RC9.0000000000000623

**Published:** 2026-09-14

**Authors:** Adriano Luis Gomes, Larissa Café Teixeira, Isabela Cristina Semensato Carloni, Alexandre Cesar Santos, Luiz G Freitas Filho

**Affiliations:** Department of Pediatric Urology, Hospital Santa Marcelina, São Paulo, Brazil

**Keywords:** congenital urogenital anomaly, Herlyn–Werner–Wunderlich syndrome, minimally invasive surgery, OHVIRA syndrome, pediatric urology, robotic surgery, uterus didelphys

## Abstract

**Introduction:**

OHVIRA (obstructed hemivagina with ipsilateral renal agenesis) syndrome, also known as Herlyn–Werner–Wunderlich syndrome, is a rare congenital anomaly of the female urogenital tract characterized by uterus didelphys, an obstructed hemivagina, and ipsilateral renal agenesis. It is typically diagnosed at puberty due to cyclic pelvic pain or hematocolpos. Early recognition in childhood is uncommon but may prevent serious complications.

**Case presentation:**

We describe a 1-year-old girl in whom OHVIRA syndrome was incidentally identified during imaging performed for unrelated findings. Ultrasonography and magnetic resonance imaging demonstrated right renal agenesis, uterine duplication, and an obstructed hemivagina. Diagnostic endoscopy confirmed vaginal duplication with a right atretic hemivagina and absence of the right ureteral orifice, suggesting an ectopic ureteral insertion. The patient underwent robotic-assisted right nephroureterectomy, partial hysterectomy, and excision of the right hemivagina without complications. She was discharged on postoperative day 2 and remains asymptomatic after 1 year of follow-up.

**Clinical discussion:**

Early identification of OHVIRA syndrome is essential to prevent complications such as infection, pelvic abscess, endometriosis, and infertility. Robotic surgery offers excellent visualization, precise dissection, and safe reconstruction, particularly in deep pelvic regions. Compared with conventional approaches, it provides reduced postoperative pain, shorter hospital stays, and faster recovery.

**Conclusion:**

Robotic-assisted surgery represents a safe and minimally invasive approach for the management of OHVIRA syndrome, even in very young patients, ensuring complete anatomical correction with minimal morbidity and rapid recovery.

## Introduction

OHVIRA (obstructed hemivagina with ipsilateral renal agenesis) syndrome, also known as Herlyn–Werner–Wunderlich syndrome, is a rare congenital anomaly of the female urogenital tract characterized by an obstructed hemivagina, uterus didelphys, and ipsilateral renal agenesis or malformation^[^1,2^]^. First described in 1922^[^3^]^, the syndrome encompasses a spectrum of clinical presentations resulting from combined Müllerian and Wolffian duct developmental anomalies.

It is most commonly diagnosed during puberty, typically presenting with dysmenorrhea, cyclic pelvic pain, and a pelvic or abdominal mass secondary to hematocolpos, or, occasionally, with a vulval bulge mimicking an imperforate hymen^[^4,5^]^.

HIGHLIGHTS

- OHVIRA (obstructed hemivagina with ipsilateral renal agenesis) syndrome is a rare congenital anomaly of the female urogenital tract.
- Early diagnosis in childhood prevents severe complications such as infection and infertility.
- Robotic-assisted surgery enables safe and precise correction of complex pelvic anatomy.
- A minimally invasive robotic approach ensures rapid recovery and minimal postoperative pain.
- This is one of the youngest reported patients with OHVIRA to be managed through robotic surgery.

Early recognition during childhood is exceptional but may prevent severe complications such as infection, pelvic abscess, endometriosis, and infertility.

This report describes a 1-year-old girl with incidentally diagnosed OHVIRA syndrome, highlighting the clinical findings and the benefits of early surgical correction using a robotic-assisted approach.

This case has been prepared in accordance with the SCARE Guidelines to ensure transparent reporting of patient history, diagnostic assessment, operative management, and follow-up outcomes^[^6^]^.

## Presentation of the case

A 1-year-old girl, weighing 11.4 kg, was referred by a pediatric endocrinologist following an incidental finding on abdominal ultrasonography performed for non-specific reasons, including relatively enlarged nipples. The examination revealed right renal agenesis, uterine and cervical duplication, and a partially septated vagina^[^7,8^]^.

Pelvic magnetic resonance imaging (MRI) demonstrated a uterus associated with a cystic, elongated structure in the right abdomen and pelvis that extended to the perineum, contained only fluid, and measured 9.7 × 5.3 × 5.1 cm, consistent with a right uterine horn and a hemivagina with incomplete septation (Figs 1 and 2).

**Figure 1.**
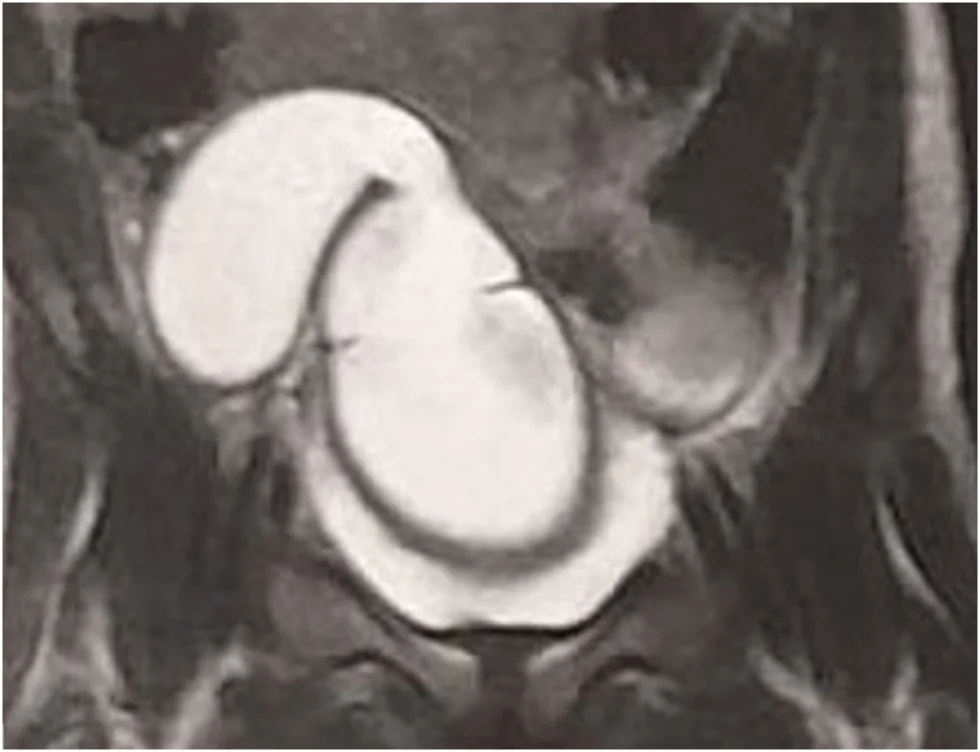
Magnetic resonance imaging (axial view) showing a cystic, elongated structure in the right abdomen and pelvis extending to the perineum, consistent with a right uterine horn and a hemivagina containing only fluid.

**Figure 2.**
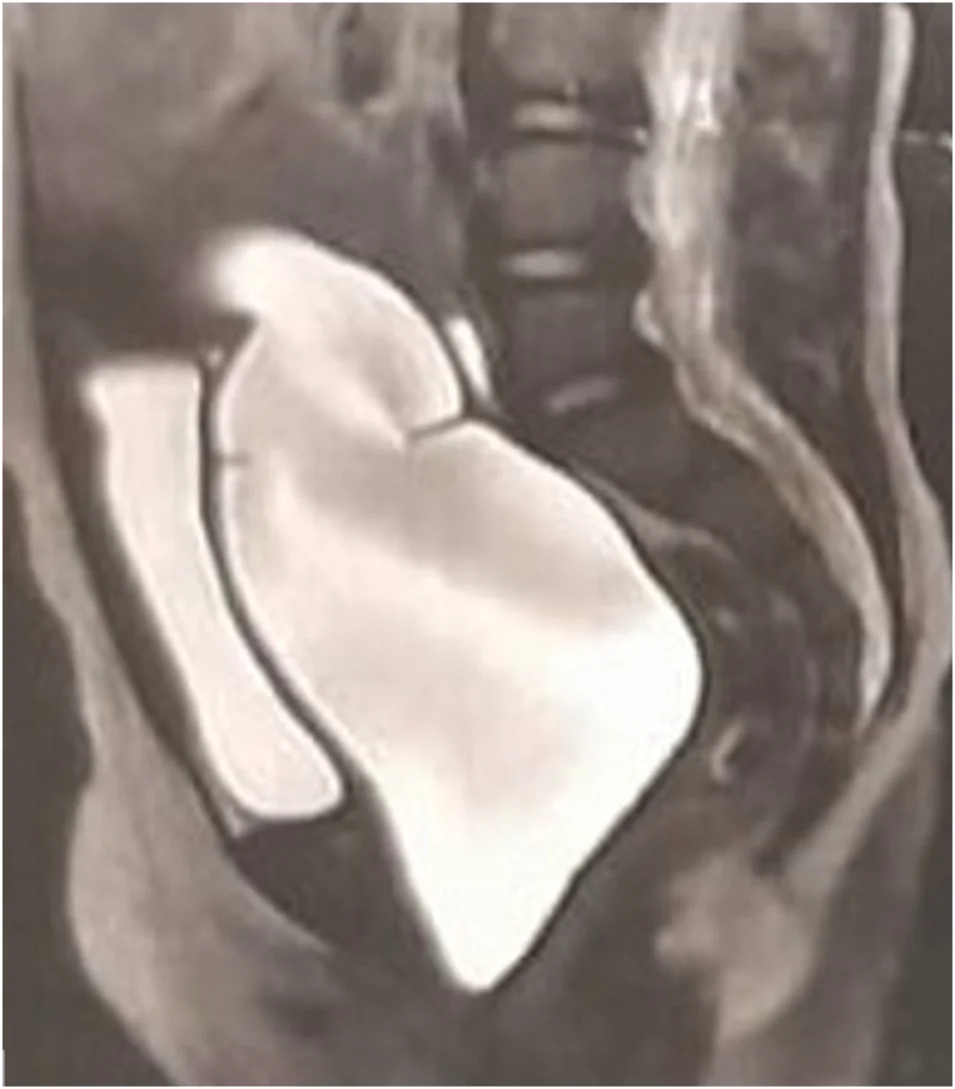
Sagittal magnetic resonance imaging confirms the presence of uterine duplication and an obstructed right hemivagina with incomplete septation, findings compatible with OHVIRA syndrome.

On physical examination, a painless, soft hypogastric mass was palpable approximately 3 cm below the umbilicus. Inspection of the external genitalia showed no evident abnormalities.

Further diagnostic evaluation was performed under general anesthesia and included genitoscopy (colposcopy) and cystoscopy to clarify the vaginal, urethral, and bladder anatomy. Endoscopy revealed bulging of the right anterolateral vaginal wall, consistent with vaginal duplication and an atretic right hemivagina. During cystoscopy, the right ureteral orifice was not identified, suggesting a possible ectopic insertion into the atretic hemivagina^[^9,10^]^.

Definitive surgical management was performed using a *robotic-assisted approach*. The patient was positioned in the dorsal decubitus position, and three 8-mm robotic trocars and one 5-mm laparoscopic trocar were introduced (Fig. 3). A right nephroureterectomy of the renal remnant, partial hysterectomy, and resection of the right hemivagina were performed without complications (Figs 4 and 5).

**Figure 3.**
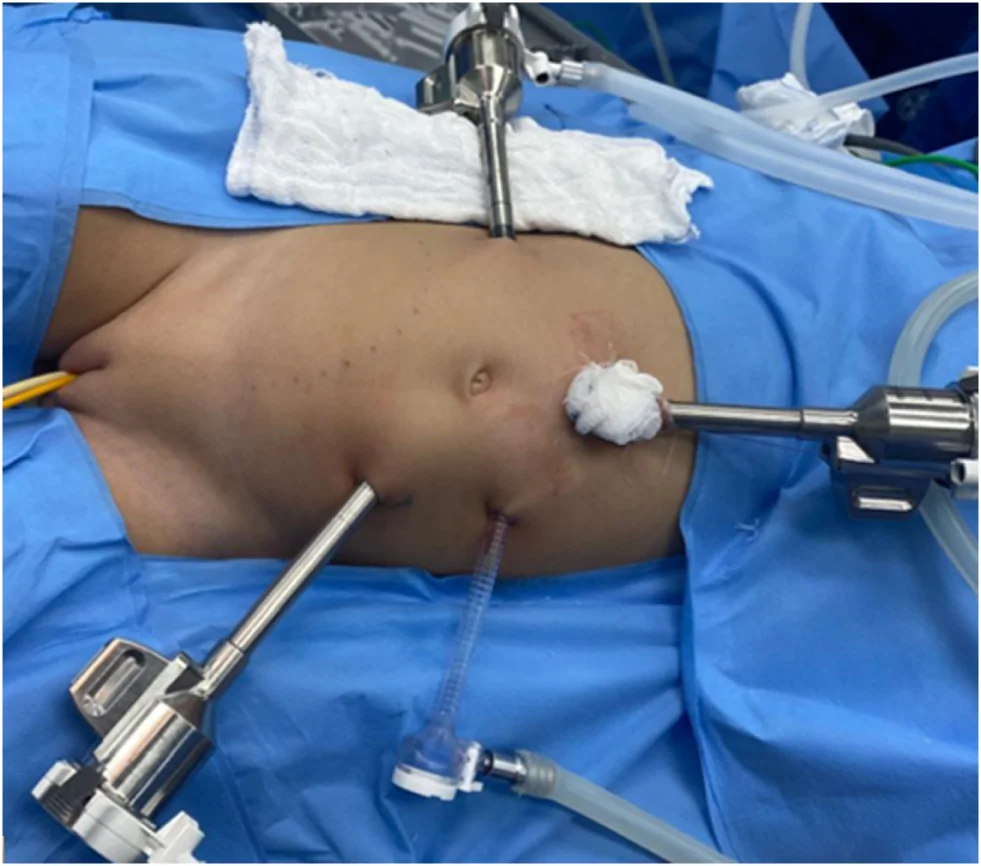
Trocar placement for the robotic-assisted procedure, showing three 8-mm robotic trocars and one 5-mm laparoscopic trocar in the dorsal decubitus position.

**Figure 4.**
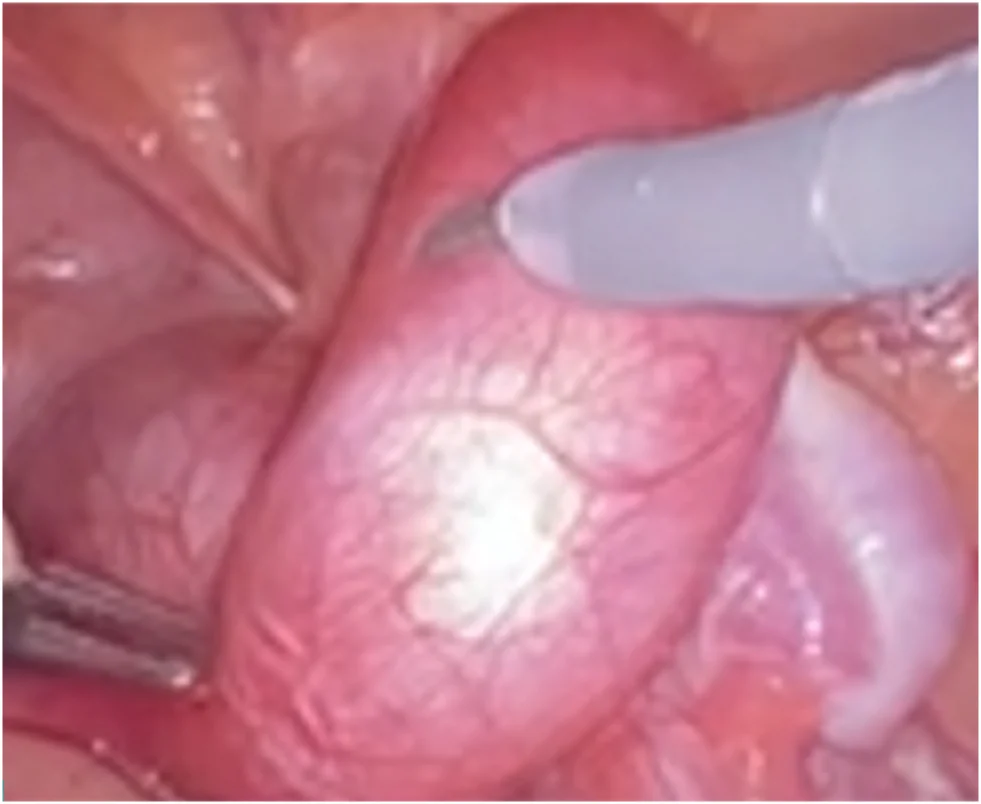
Intraoperative view during robotic-assisted right nephroureterectomy and partial hysterectomy, demonstrating precise dissection of the pelvic structures.

**Figure 5.**
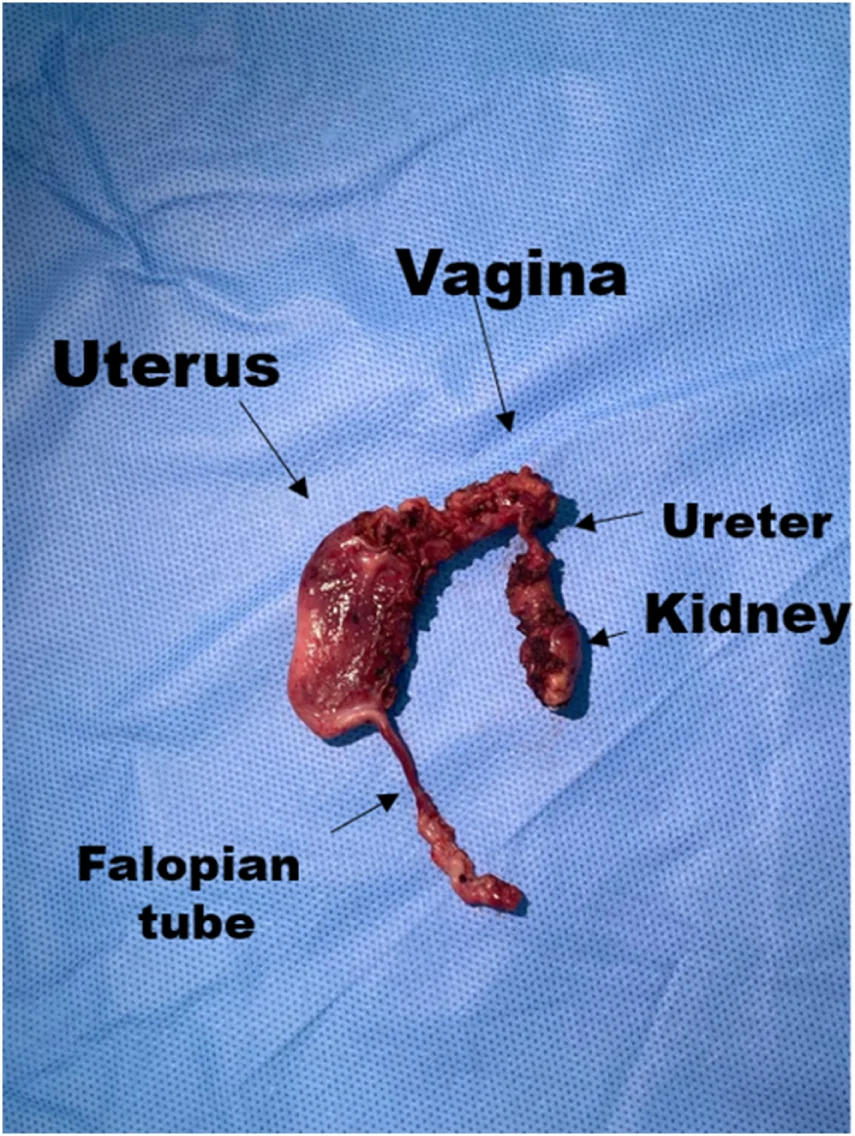
Specimen following robotic-assisted resection, including the right renal remnant, ureter, and obstructed hemivagina.

The postoperative period was uneventful. The patient was discharged on postoperative day 2 and remains asymptomatic after 1 year of follow-up^[^11,12^]^.

## Discussion

OHVIRA syndrome is a rare congenital malformation of the female urogenital tract that results from a combination of Müllerian and Wolffian duct anomalies. Because of its rarity, no formal screening recommendations exist; therefore, early recognition relies on clinical vigilance and imaging evaluation, particularly in patients with renal agenesis or other urogenital abnormalities^[^13,14^]^.

The syndrome typically presents during adolescence with dysmenorrhea, cyclical pelvic pain, or a pelvic mass caused by hematocolpos. However, presentation in infancy or early childhood is uncommon and usually incidental, as in the present case. Symptoms beyond menstrual abnormalities may include recurrent abdominal pain, urinary incontinence or retention^[^13–15^]^.

Renal agenesis is the most frequently associated anomaly, followed by ureteric duplication, ectopic ureteric insertion, renal dysplasia, or multicystic kidney. Gynecological malformations often include uterus didelphys and vaginal duplication^[^15–18^]^. In children, these anomalies are usually asymptomatic and are often identified incidentally during imaging for unrelated conditions.

Early diagnosis is crucial to prevent acute and chronic complications, such as pelvic abscess, vesicovaginal fistula, endometriosis, pelvic inflammatory disease, and future infertility^[^1,12,18^]^. Physical examination in prepubertal patients may reveal a palpable abdominal mass, whereas postmenarchal patients typically present with recurrent lower abdominal pain or vulvar bulging^[^13–15^]^.

Imaging studies play a fundamental role in diagnosis. Abdominal and pelvic ultrasonography are valuable first-line tools, allowing detection of renal agenesis and cystic pelvic lesions. Computed tomography or MRI provides superior anatomical detail and should be complemented by endoscopic assessment, including colposcopy and cystoscopy, to delineate the anatomy of the vagina, uterus, urethra, bladder, and ureters^[^16–19^]^. This multimodal approach is essential for accurate diagnosis and optimal surgical planning.

Surgical treatment aims to relieve obstruction, restore normal anatomy, and preserve reproductive potential. In symptomatic cases, hysteroscopic incision of the vaginal septum can provide immediate decompression. In more complex presentations or when associated renal anomalies are present, conventional or laparoscopic hysterectomy and nephrectomy may be indicated^[^19–23^]^.

Robotic surgery has recently emerged as a valuable alternative in pediatric and gynecologic procedures. It offers enhanced three-dimensional visualization, precise dissection, and improved ergonomics, particularly in deep pelvic regions. These advantages facilitate safe resections and reconstructions with minimal risk of injury to adjacent structures^[^13^]^. Furthermore, robotic-assisted approaches are associated with less postoperative pain, faster recovery, and shorter hospital stays compared with open or conventional laparoscopic techniques^[^24^]^.

The present case demonstrates that robotic surgery can be safely performed even in very young patients, providing complete anatomical correction with minimal morbidity. Early surgical intervention with robotic assistance may prevent long-term complications and ensure optimal functional and reproductive outcomes.

## Strengths, weaknesses, and limitations

The main strength of this report is the detailed clinical, imaging, and surgical documentation of a rare condition, providing valuable insights into diagnosis and management. However, as a single case, it does not allow for generalization of outcomes or comparisons between surgical approaches. Further case series and long-term follow-up studies are needed to validate the proposed treatment strategy.

## Conclusion

OHVIRA syndrome is a rare congenital anomaly with diverse clinical presentations and diagnostic challenges, particularly in prepubertal children. Early recognition is essential to prevent long-term complications and preserve reproductive potential. Among the available surgical options, robotic-assisted surgery represents an excellent minimally invasive alternative, enabling precise dissection, rapid postoperative recovery, and an early return to normal activities, even in very young patients.

## Data Availability

Data availability is not applicable to this article as no datasets were generated or analyzed during the current study.

## Patient perspective

The patient’s parents expressed satisfaction with the surgical outcome and follow-up, highlighting the rapid recovery and absence of postoperative complications.
